# Moderating effect of cardiorespiratory fitness on sickness absence in occupational groups with different physical workloads

**DOI:** 10.1038/s41598-023-50154-9

**Published:** 2023-12-21

**Authors:** Daniel Väisänen, Peter J. Johansson, Lena Kallings, Erik Hemmingsson, Gunnar Andersson, Peter Wallin, Sofia Paulsson, Teresia Nyman, Andreas Stenling, Magnus Svartengren, Elin Ekblom-Bak

**Affiliations:** 1 Department of Physical Activity and Health, The Swedish School of Sport and Health Sciences, Stockholm, Sweden; 2 Department of Medical Sciences, Occupational and Environmental Medicine, Uppsala University, Uppsala, Sweden; 3 Occupational and Environmental Medicine, Uppsala University Hospital, Uppsala, Sweden; 4 Department of Research, HPI Health Profile Institute, Danderyd/Stockholm, Sweden; 5 Department of Psychology, Umeå University, Umeå, Sweden; 6 Department of Sport Science and Physical Education, University of Agder, Kristiansand, Norway

**Keywords:** Cardiovascular diseases, Psychiatric disorders, Diseases, Health occupations

## Abstract

Sickness absence from work has a large adverse impact on both individuals and societies in Sweden and the costs for sickness absence were calculated to 64.6 billion Swedish kronor (approx. 5.6 billion in Euros) in 2020. Although high cardiorespiratory fitness may protect against potential adverse effects of high physical workload, research on the moderating effect of respiratory fitness in the relation between having an occupation with high physical workload and sickness absence is scarce. To study the moderating effect of cardiorespiratory fitness in the association between occupation and psychiatric, musculoskeletal, and cardiorespiratory diagnoses. Data was retrieved from the HPI Health Profile Institute database (1988–2020) and Included 77,366 participants (mean age 41.8 years, 52.5% women) from the Swedish workforce. The sample was chosen based on occupational groups with a generally low education level and differences in physical workload. Hurdle models were used to account for incident sickness absence and the rate of sickness absence days. There were differences in sickness absence between occupational groups for musculoskeletal and cardiorespiratory diagnoses, but not for psychiatric diagnoses. In general, the association between occupation and musculoskeletal and cardiorespiratory diagnoses was moderated by cardiorespiratory fitness in most occupational groups with higher physical workload, whereas no moderating effect was observed for psychiatric diagnoses. The study results encourage community and workplace interventions to both consider variation in physical workload and to maintain and/or improve cardiorespiratory fitness for a lower risk of sickness absence, especially in occupations with high physical workload.

## Introduction

Sickness absence has a large impact on both the individual and societal level. In Sweden, the costs for sickness absence were calculated to 64.6 billion Swedish kronor (approx. 5.6 billion in Euros) in 2020^1^. However, the underlying mechanisms are still poorly understood. Some of these mechanisms appear to be directly associated with the work situation and type of occupation^2–4^, whereas others appear to be structural and related to organizational, social policy, and economic factors^5–7^. Also, differences in lifestyle between occupational groups may explain some of the variation, with overweight/obesity and low levels of leisure time physical activity being more prevalent in blue-collar and low-skilled occupational groups^8–10^.

Another important factor for sickness absence may be the level of physical activity at work. Whereas a wealth of evidence indicate that leisure-time physical activity has positive health effects^11^, adverse associations between high occupational physical activity (physical workload) and various health outcomes^12,13^ and sickness absence^10,14–16^ have been reported in previous studies. One factor that may moderate these adverse associations is cardiorespiratory fitness (CRF)^17^. CRF is the capacity of the circulatory and respiratory systems to supply oxygen to skeletal muscle mitochondria for energy production needed during physical activity^18^. Active workers with higher CRF will experience a lower relative workload for the same absolute load, and therefore hypothetically have a lower relative strain on the cardiovascular system^19^. Important determinants of CRF are physical activity, body mass index (BMI), genetics, age, and sex^20,21^, of which only physical activity and BMI are modifiable.

Although CRF is a potent marker of physical and mental health, studies on the link between CRF and sickness absence are scarce. In cross-sectional studies, low CRF has been associated with short-term sickness absence in Finish military personnel and Swedish office workers^22,23^. A Norwegian study found that lower CRF in conscripts were moderately associated with higher musculoskeletal sickness absence 5–15 years later^24^. However, no previous study has examined the association between occupational groups, CRF, and sickness absence, and whether a higher CRF may attenuate the commonly reported increased risk of sickness absence in occupations with high physical workload. Identifying occupational groups that would gain from improvement in CRF would provide important knowledge for targeted, preventive efforts for a sustainable working life.

Hence, the aims of the present study were (a) to investigate the association between occupational groups with different physical workload and sickness absence related to psychiatric, musculoskeletal, and cardiorespiratory diagnoses (b) to assess whether CRF moderates any of the associations. To minimize the influence of variation in socioeconomics between occupations, we chose to include only occupational groups with low educational requirements in the analyses.

## Results

Age, smoking habits, CRF level, and proportion of women varied between occupational groups (Table 1). A total of 23,784 participants (31%) had at least one spell of sickness absence after the HPA due to any of the studied diagnoses, resulting in 4,852,589 sickness absence days during a median follow-up of 10.8 years. A total of *n* = 7531 had a sickness absence spell in more than one of the three diagnoses and n = 1257 participants in all three diagnoses. The number of sickness absence days after an HPA were highest for psychiatric (2,334,420) diagnoses followed by musculoskeletal (2,111,453), and cardiorespiratory (406,715) diagnoses. First time sickness absence incidence after the HPA was highest for musculoskeletal (13,849 days), followed by psychiatric (10,754 days), and cardiorespiratory (5,861 days) diagnoses.

**Table 1:** Characteristics of occupational groups.

	Office assistants	Mobile plant operators and vehicle drivers	Shop staff	Heavy truck and lorry drivers	Assemblers, machine operators and related	Assistant nurses and home care	Cleaners	Construction craftsmen and related trades	Construction workers
n	20,014	3470	6407	1792	15,067	13,707	4193	7385	5331
Age, mean (SD)	43 (11)	42 (12)	37 (12)	41 (12)	41 (11)	43 (11)	45 (11)	42 (12)	39 (12)
Women	85%	8%	60%	5%	15%	92%	90%	8%	2%
Short education	70%	91%	81%	94%	90%	89%	92%	93%	94%
Daily smoking	11%	15%	14%	15%	13%	20%	24%	13%	14%
Self-reported physical workload from HPA questionnaire									
Mostly sedentary	90%	66%	42%	50%	34%	17%	9%	14%	10%
Physically active	8%	20%	36%	25%	41%	45%	50%	43%	30%
Occasionally physically demanding	2%	12%	19%	21%	22%	34%	35%	38%	46%
Occasionally very physically demanding	0%	2%	3%	3%	3%	4%	6%	5%	15%
Self-reported physical workload from Statistics Sweden									
Streneous work	3%	28%	22%	40%	17%	28%	32%	39%	60%
Physical workload in METs from Tudor-Locke et al.^25^									
MET estimates	1.8	2.7	2.0	2.7	2.7	2.5	3.6	4.3	4.3
Cardiorespiratory fitness ml·min^−1^ kg^−1^, mean (SD)	35.7 (10)	34.4 (9.6)	37.4 (10.3)	34.4 (9.3)	35.4 (9.6)	33.7 (9.6)	32.8 (9.1)	35.9 (9.7)	37.2 (9.8)
Cardiorespiratory fitness groups									
Low < 32 ml·min^−1^ kg^−1^	40%	45%	32%	43%	40%	48%	52%	38%	33%
Medium 32–42 ml·min^−1^ kg^−1^	36%	35%	38%	37%	37%	34%	33%	38%	39%
High > 42 ml·min^−1^ kg^−1^	25%	20%	30%	19%	23%	18%	15%	24%	28%
Sickness absence days by cause									
Psychiatric, mean (SD)	29 (145)	16 (114)	29 (145)	13 (119)	17 (122)	43 (202)	35 (196)	16 (116)	13 (109)
Musculoskeletal, mean (SD)	11 (79)	22 (123)	22 (129)	17 (79)	21 (108)	41 (174)	45 (187)	23 (106)	23 (103)
Cardiorespiratory, mean (SD)	3 (47)	7 (59)	3 (39)	3 (28)	4 (54)	6 (60)	5 (53)	6 (59)	4 (51)
Percentage with a sickness absence diagnosis after the HPA by cause									
Psychiatric	14%	8%	15%	6%	8%	18%	13%	8%	7%
Musculoskeletal	9%	16%	13%	13%	16%	22%	22%	17%	18%
Cardiorespiratory	5%	7%	6%	5%	6%	10%	9%	7%	6%

Of the total study sample, 69% did not have any sickness absence > 14 days. In the different occupational groups, the proportions of zero‐absences (descending order) were 78% in heavy truck and lorry drivers, 74% in office assistants, 72% in assemblers, machine operators and related, 72% in mobile plant operators and vehicle drivers, 71% in construction craftsmen and related trades, 71% in construction workers, 70% in shop staff, 60% in cleaners, and 53% in assistant nurses and home care.

### Occupational groups and sickness absence

**Figure 1:**
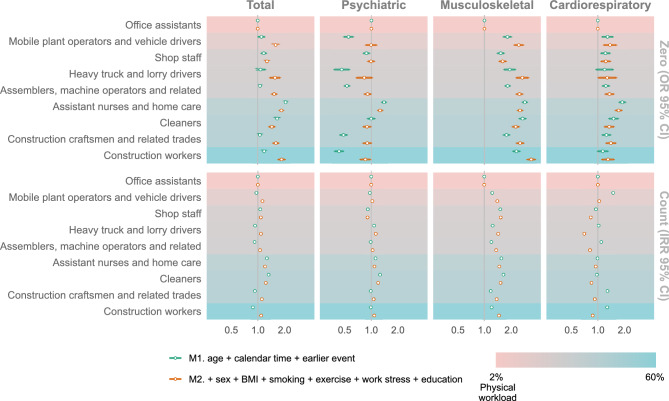
Association between occupational groups and incident sickness absence (as odds ratio, 95% CI), as well as rate of sickness absence days in those with sickness absence (as incident rate ratio, 95% CI).

Total incident sickness absence was in general higher in occupations with higher occupational physical workload (Fig. 1, upper part). Incident sickness absence due to musculoskeletal and cardiorespiratory causes displayed similar associations as for the total sickness absence outcome, while incident sickness absence due to psychiatric causes was in general lower in more physically demanding occupations. Rate of sickness absence days in those with sickness absence displayed less variation between the occupational groups, with only a clear trend of higher numbers of sickness absence days due to musculoskeletal causes with higher occupational workload (Fig. 1, lower part).

### Moderating effect of cardiorespiratory fitness

**Figure 2:**
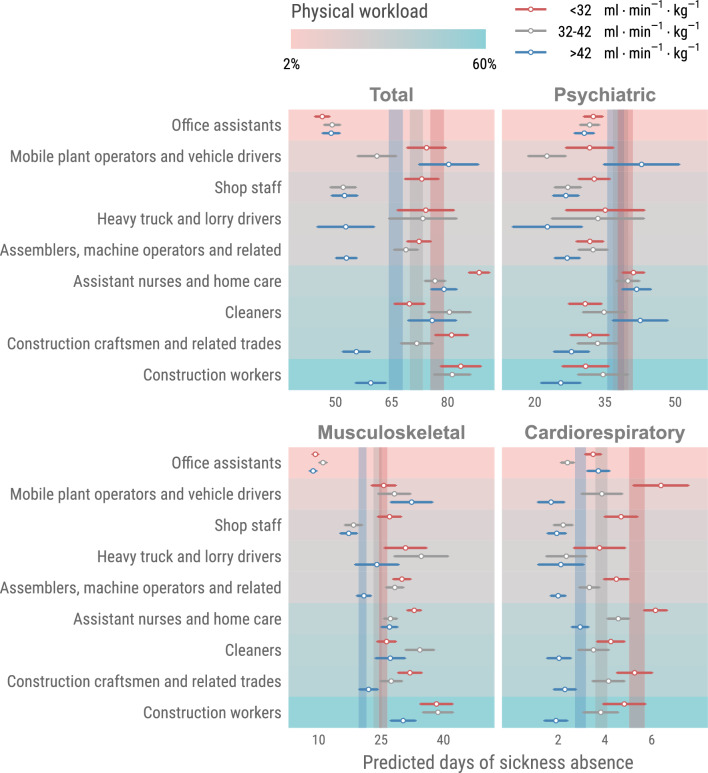
Analyses are adjusted for age, sex, BMI, smoking, exercise, work stress, education, earlier event, and calendar time. Colored vertical bars represent confidence intervals for the CRF-groups as a main model for each sickness absence diagnose and total sickness absence.

The moderating effect of CRF on the relationship between different occupational groups and both the risk and rate of sickness absence are presented in Tables 2 and 3. Figure 2, shows that within specific occupational groups—namely, *Shop staff, Heavy truck and lorry drivers, Assemblers, machine operators and related, Assistant nurses and home care, Construction craftsmen and related, and Construction workers*—higher CRF significantly moderated the occupational impact on total sickness absence days. Conversely, in *Office assistants, Mobile plant operators and vehicle drivers,* and *Cleaners*, higher CRF did not exhibit a moderating effect on occupation-related sickness absence This moderating influence of CRF was consistent across these occupational groups for sickness days attributed to musculoskeletal causes. When examining sickness absence due to cardiorespiratory causes, CRF played a strong moderating role across all occupational groups, except for *Office assistants*, which represent the occupational group with the lowest physical workload. For psychiatric-related sickness absence days, the moderating effect of higher CRF was only observed in *Shop staff* and *Assemblers*, *machine operators and related*."

**Table 2:** Odds ratio 95% CI from a binary logit model where the risk of not having sickness absence is modeled.

	VO_2_max (ml/kg/min)	N	Total		Psychiatric		Musculoskeletal		Cardiorespiratory	
			M1	M2	M1	M2	M1	M2	M1	M2
Office assistants	< 32	15,816	1.00	1.00	1.00	1.00	1.00	1.00	1.00	1.00
	32–42	12,230	0.94 (0.87–1.02)	1.06 (0.97–1.15)	0.95 (0.86–1.05)	1.05 (0.95–1.17)	0.97 (0.87–1.08)	1.10 (0.97–1.24)	0.77 (0.67–0.89)	0.88 (0.76–1.03)
	> 42	11,982	**0.80 (0.73–0.87)**	0.98 (0.88–1.08)	**0.85 (0.77–0.95)**	1.03 (0.91–1.16)	**0.79 (0.69–0.90)**	0.97 (0.84–1.13)	**0.68 (0.58–0.79)**	0.85 (0.71–1.02)
Mobile plant operators and vehicle drivers	< 32	3,120	1.00	1.00	1.00	1.00	1.00	1.00	1.00	1.00
	32–42	2,100	0.88 (0.73–1.06)	0.93 (0.76–1.14)	0.99 (0.74–1.33)	0.96 (0.71–1.32)	0.89 (0.71–1.11)	0.96 (0.76–1.22)	0.89 (0.67–1.19)	0.98 (0.72–1.32)
	> 42	1,720	**0.79 (0.63–0.98)**	0.87 (0.68–1.12)	0.89 (0.65–1.24)	0.88 (0.60–1.27)	0.87 (0.67–1.12)	0.98 (0.73–1.32)	**0.49 (0.33–0.74)**	**0.58 (0.37–0.89)**
Shop staff	< 32	4,132	1.00	1.00	1.00	1.00	1.00	1.00	1.00	1.00
	32–42	4,002	**0.85 (0.73–0.99)**	0.87 (0.75–1.02)	0.91 (0.76–1.09)	0.92 (0.76–1.12)	0.88 (0.73–1.06)	0.94 (0.78–1.15)	**0.73 (0.57–0.93)**	**0.76 (0.59–0.97)**
	> 42	4,680	**0.73 (0.63–0.86)**	**0.77 (0.65–0.92)**	**0.79 (0.66–0.95)**	0.83 (0.67–1.03)	**0.79 (0.65–0.97)**	0.89 (0.71–1.11)	**0.56 (0.43–0.74)**	**0.60 (0.45–0.82)**
Heavy truck and lorry drivers	< 32	1,552	1.00	1.00	1.00	1.00	1.00	1.00	1.00	1.00
	32–42	1,150	0.87 (0.65–1.16)	1.02 (0.74–1.41)	0.71 (0.44–1.13)	0.69 (0.41–1.15)	1.02 (0.73–1.43)	1.24 (0.86–1.81)	0.98 (0.61–1.59)	1.15 (0.67–1.95)
	> 42	882	0.78 (0.56–1.09)	1.01 (0.69–1.50)	0.63 (0.37–1.05)	0.60 (0.33–1.10)	**0.93 (0.63–1.37)**	1.30 (0.82–2.05)	0.81 (0.45–1.48)	1.06 (0.53–2.09)
Assemblers, machine operators and related	< 32	12,106	1.00	1.00	1.00	1.00	1.00	1.00	1.00	1.00
	32–42	9,544	0.96 (0.88–1.05)	1.04 (0.95–1.15)	0.91 (0.79–1.04)	0.99 (0.85–1.15)	**0.97 (0.87–1.07)**	1.06 (0.95–1.19)	0.94 (0.81–1.08)	1.03 (0.88–1.19)
	> 42	8,484	**0.83 (0.75–0.92)**	0.95 (0.85–1.06)	**0.85 (0.73–0.99)**	0.96 (0.81–1.14)	**0.89 (0.79–1.00)**	1.03 (0.90–1.17)	**0.65 (0.55–0.78)**	**0.76 (0.62–0.92)**
Assistant nurses and home care	< 32	13,062	1.00	1.00	1.00	1.00	1.00	1.00	1.00	1.00
	32–42	8,080	**0.89 (0.82–0.97)**	0.99 (0.90–1.08)	1.02 (0.93–1.13)	1.08 (0.97–1.21)	0.92 (0.84–1.01)	1.04 (0.94–1.14)	**0.74 (0.66–0.84)**	**0.85 (0.75–0.97)**
	> 42	6,272	**0.71 (0.64–0.78)**	**0.85 (0.76–0.95)**	**0.82 (0.73–0.92)**	0.90 (0.79–1.02)	**0.75 (0.67–0.84)**	0.92 (0.81–1.04)	**0.67 (0.58–0.77)**	**0.84 (0.71–0.98)**
Cleaners	< 32	4,362	1.00	1.00	1.00	1.00	1.00	1.00	1.00	1.00
	32–42	2,380	0.99 (0.85–1.16)	1.08 (0.92–1.27)	1.06 (0.86–1.31)	1.15 (0.92–1.44)	1.11 (0.94–1.31)	1.22 (1.02–1.46)	0.84 (0.67–1.06)	0.89 (0.69–1.13)
	> 42	1,644	0.84 (0.70–1.01)	1.01 (0.82–1.25)	1.13 (0.89–1.44)	1.35 (1.04–1.77)	0.83 (0.67–1.02)	1.00 (0.79–1.26)	**0.64 (0.48–0.87)**	0.73 (0.52–1.01)
Construction craftsmen and related trades	< 32	5,616	1.00	1.00	1.00	1.00	1.00	1.00	1.00	1.00
	32–42	4,704	0.93 (0.82–1.06)	1.07 (0.94–1.22)	1.03 (0.84–1.27)	1.14 (0.91–1.42)	0.95 (0.82–1.10)	1.10 (0.95–1.29)	0.85 (0.70–1.04)	0.98 (0.80–1.20)
	> 42	4,450	**0.84 (0.73–0.96)**	1.07 (0.91–1.25)	0.92 (0.74–1.15)	1.11 (0.86–1.44)	0.91 (0.77–1.08)	1.18 (0.98–1.42)	**0.58 (0.45–0.74)**	**0.74 (0.57–0.97)**
Construction workers	< 32	3,496	1.00	1.00	1.00	1.00	1.00	1.00	1.00	1.00
	32–42	3,454	1.06 (0.90–1.24)	1.17 (0.99–1.38)	0.88 (0.68–1.15)	0.91 (0.69–1.20)	1.08 (0.91–1.29)	1.19 (0.99–1.43)	0.91 (0.71–1.18)	1.05 (0.80–1.37)
	> 42	3,712	**0.83 (0.70–0.98)**	0.96 (0.80–1.17)	**0.66 (0.50–0.88)**	0.73 (0.53–1.00)	0.94 (0.77–1.14)	1.08 (0.87–1.34)	**0.51 (0.37–0.71)**	**0.64 (0.44–0.92)**

Zero part of a hurdle model those having/not having sickness absence- M1 is controlled for age, event before and calendar time. M2 is additionally controlled for diet, exercise, BMI, smoking, work stress and education. Significant values are in bold.

**Table 3:** Incident rate ratio with 95% CI from negative binomial models where the amount of sickness absence days after the HPA is modelled in those with sickness absence.

	VO_2_max (ml/kg/min)	Total			Psychiatric			Musculoskeletal			Cardiorespiratory		
		N	M1	M2	N	M1	M2	N	M1	M2	N	M1	M2
Office assistants	< 32	2,114	1.00	1.00	1,118	1.00	1.00	902	1.00	1.00	581	1.00	1.00
	32–42	1,653	0.95 (0.94–0.95)	0.98 (0.98–0.99)	988	0.93 (0.93–0.94)	0.97 (0.96–0.98)	655	1.05 (1.04–1.06)	1.08 (1.07–1.09)	345	0.71 (0.70–0.72)	0.71 (0.70–0.73)
	> 42	1,505	0.92 (0.91–0.92)	0.98 (0.97–0.98)	1,012	0.91 (0.90–0.91)	0.98 (0.97–0.99)	505	0.86 (0.85–0.87)	0.90 (0.89–0.92)	292	1.05 (1.04–1.07)	1.09 (1.07–1.12)
Mobile plant operators and vehicle drivers	< 32	459	1.00	1.00	125	1.00	1.00	294	1.00	1.00	139	1.00	1.00
	32–42	294	0.81 (0.80–0.82)	0.86 (0.85–0.87)	98	0.65 (0.63–0.66)	0.70 (0.69–0.72)	179	1.09 (1.07–1.11)	1.18 (1.16–1.20)	85	0.60 (0.58–0.62)	0.64 (0.62–0.66)
	> 42	217	1.08 (1.06–1.09)	1.14 (1.12–1.16)	89	1.11 (1.09–1.13)	1.10 (1.08–1.13)	130	1.19 (1.17–1.22)	1.37 (1.34–1.40)	36	0.42 (0.40–0.44)	0.45 (0.43–0.48)
Shop staff	< 32	690	1.00	1.00	328	1.00	1.00	361	1.00	1.00	192	1.00	1.00
	32–42	594	0.74 (0.73–0.74)	0.77 (0.76–0.78)	329	0.84 (0.83–0.85)	0.86 (0.85–0.87)	284	0.67 (0.66–0.68)	0.76 (0.75–0.77)	125	0.67 (0.64–0.69)	0.61 (0.59–0.64)
	> 42	621	0.77 (0.76–0.77)	0.86 (0.86–0.87)	381	0.88 (0.87–0.89)	0.93 (0.92–0.94)	269	0.64 (0.63–0.65)	0.85 (0.84–0.86)	105	0.65 (0.62–0.68)	0.70 (0.67–0.73)
Heavy truck and lorry drivers	< 32	183	1.00	1.00	52	1.00	1.00	117	1.00	1.00	47	1.00	1.00
	32–42	125	1.08 (1.06–1.10)	1.09 (1.07–1.11)	36	1.27 (1.23–1.31)	1.19 (1.15–1.23)	81	1.14 (1.11–1.17)	1.42 (1.38–1.47)	30	0.62 (0.58–0.66)	0.86 (0.80–0.93)
	> 42	94	0.85 (0.83–0.87)	0.96 (0.93–0.99)	30	1.00 (0.97–1.04)	1.09 (1.05–1.14)	59	0.82 (0.79–0.84)	1.13 (1.08–1.17)	19	0.60 (0.56–0.65)	0.78 (0.71–0.86)
Assemblers, machine operators and related	< 32	1,772	1.00	1.00	518	1.00	1.00	1,132	1.00	1.00	504	1.00	1.00
	32–42	1,375	0.88 (0.87–0.88)	0.94 (0.93–0.94)	448	0.99 (0.98–1.00)	1.01 (1.00–1.02)	852	0.86 (0.85–0.87)	0.91 (0.90–0.92)	363	0.70 (0.69–0.71)	0.79 (0.78–0.80)
	> 42	1,094	0.71 (0.70–0.71)	0.78 (0.78–0.79)	428	0.83 (0.82–0.84)	0.84 (0.83–0.85)	672	0.64 (0.63–0.65)	0.70 (0.69–0.71)	210	0.57 (0.56–0.59)	0.69 (0.67–0.71)
Assistant nurses and home care	< 32	3,140	1.00	1.00	1,348	1.00	1.00	1,971	1.00	1.00	918	1.00	1.00
	32–42	1,904	0.81 (0.81–0.82)	0.83 (0.82–0.83)	987	0.84 (0.84–0.85)	0.86 (0.85–0.86)	1,133	0.76 (0.75–0.76)	0.77 (0.76–0.77)	438	0.87 (0.86–0.89)	0.80 (0.79–0.81)
	> 42	1,340	0.86 (0.86–0.86)	0.88 (0.88–0.89)	747	0.96 (0.95–0.96)	0.98 (0.97–0.98)	722	0.79 (0.78–0.79)	0.81 (0.80–0.81)	303	0.55 (0.54–0.56)	0.49 (0.48–0.50)
Cleaners	< 32	855	1.00	1.00	281	1.00	1.00	579	1.00	1.00	239	1.00	1.00
	32–42	503	0.98 (0.97–0.99)	1.04 (1.03–1.05)	202	0.88 (0.87–0.89)	0.95 (0.94–0.96)	354	1.00 (0.99–1.01)	1.03 (1.02–1.04)	121	0.78 (0.76–0.80)	0.78 (0.76–0.81)
	> 42	330	0.82 (0.81–0.83)	0.92 (0.91–0.93)	175	0.79 (0.78–0.80)	0.96 (0.94–0.97)	201	0.80 (0.79–0.81)	0.85 (0.84–0.86)	69	0.47 (0.45–0.49)	0.46 (0.44–0.48)
Construction craftsmen and related trades	< 32	886	1.00	1.00	219	1.00	1.00	566	1.00	1.00	285	1.00	1.00
	32–42	685	0.90 (0.89–0.90)	0.95 (0.94–0.96)	214	1.07 (1.05–1.08)	1.16 (1.14–1.17)	424	0.80 (0.79–0.81)	0.82 (0.81–0.83)	189	0.86 (0.85–0.88)	0.86 (0.85–0.88)
	> 42	574	0.72 (0.72–0.73)	0.81 (0.80–0.82)	203	0.98 (0.97–0.99)	1.20 (1.18–1.22)	357	0.60 (0.60–0.61)	0.63 (0.62–0.64)	110	0.63 (0.61–0.65)	0.65 (0.63–0.67)
Construction workers	< 32	511	1.00	1.00	124	1.00	1.00	350	1.00	1.00	143	1.00	1.00
	32–42	541	0.89 (0.88–0.90)	0.92 (0.91–0.93)	140	1.02 (1.00–1.04)	1.12 (1.09–1.14)	367	0.86 (0.85–0.87)	0.92 (0.90–0.93)	121	0.85 (0.82–0.87)	0.84 (0.81–0.86)
	> 42	477	0.69 (0.68–0.70)	0.70 (0.69–0.70)	134	0.86 (0.85–0.88)	0.88 (0.86–0.90)	333	0.68 (0.67–0.69)	0.71 (0.70–0.72)	65	0.76 (0.73–0.79)	0.83 (0.79–0.87)

Count part of a hurrdle model number of days of sickness absence for those who has sickness absence- M1 is controlled for age, event before and calendar time. M2 is additionally controlled for diet, exercise, BMI, smoking, work stress and education.

## Discussion

The main findings in this large sample of Swedish workers (mainly low educated occupational groups) were that (1) first time sickness absence after an HPA varied according to occupational physical workload, (2) in general, while occupational groups with higher physical workload had higher risk for total sickness absence and sickness absence due to musculoskeletal and cardiorespiratory causes, the risk was lower for sickness absence due to psychiatric causes in occupations with the highest occupational physical activity, also, *Assistant nurses and home care* had the highest risk in psychiatric diagnoses irrespective of adjustment, (3) predicted days of sickness absence due to cardiorespiratory causes were consistently lower in workers with higher CRF in occupations with higher physical workload, 4) predicted days of sickness absence due to musculoskeletal causes were generally but not consistently lower in workers with higher CRF, (5) there was no clear moderating effect of CRF for predicted days of sickness absence due to psychiatric causes.

In the present study, *Assistant nurses and home care* and *Cleaners* had the highest total incidence in sickness absence in the least adjusted models, while *Assistant nurses and home care* and *Constructions workers* had highest total sickness absence in the fully adjusted model*.* This is comparable to a Danish study, which showed high prevalence of sickness absence in Kindergarten teachers, people employed in Day care, Health care, Janitorial work, Food preparation, Social service sectors and Unskilled workers^26^. These occupational groups have previously been associated with high emotional demands, which has been reported as a risk factor for sickness absence^27^. Moreover, occupations with higher physical workload has been linked to higher sickness absence, for instance high physical workload has been attributed to 25% of sickness absence cases in Norway and Denmark^28,29^. Importantly, increased age has been associated to greater negative consequences of high physical workload^30^, suggested to be caused by generally lower physical capacity among older compared to younger workers^17^. In the current study, the associations varied somewhat depending on the diagnosis attributed to sickness absence, and so did also the possible moderating effect of CRF in the associations (discussed further below).

**Sickness absence due to psychiatric diagnoses** accounted for the largest part of sickness absence in the present study with a generally early life onset^31^. In the current study, *Assistance nurses and home care* had highest risk of all groups in both models. In the least adjusted model, we saw large differences between occupational groups with differing composition of sex. Occupational groups with a greater proportion of women (for example O*ffice assistants)* had in general higher risk of psychiatric sickness absence, which has been reported previously^32^. However, in multivariable adjusted models (adjusting for sex), the associations were attenuated. Further, in a Swedish register based study in 19–29 year old, sickness absence risk due to common mental disorders was slightly higher for manual workers compared to non-manual workers, and those employed in education and health and social services^33^. The differences may be attributed to differences in socioeconomic strata between studies. Also, a Swedish population-based study showed that public sector employees and occupational groups requiring higher (post-secondary) education had higher mental sickness absence, as well had personal care workers, travel attendants, and other service workers^34^. In addition to there being no observed effect of physical workload in the present study, no clear trends were observed in the moderating effect of CRF on the association between occupation and sickness absence. Previous research has indicated a lower risk of sickness absence due to mental health diagnoses with higher CRF.^22,23,35^. The observed differences between studies are likely related to socioeconomic differences or other factors related to CRF as the present study sample consists of occupations requiring mainly low education.

**Sickness absence due to musculoskeletal diagnoses** are characterized by impairments in the muscles, bones, joints, and adjacent connective tissue and has a strong association to physical workload, in terms of heavy lifting, awkward postures, and repetitive movements^36^. In the present study, there was a clear increased risk for sickness absence due to musculoskeletal disorders in all occupations with any type of physical demands compared to office assistants. Studies are sparse on musculoskeletal sickness absence in different occupational groups. However, it is reported that musculoskeletal diagnoses follow a steep socioeconomic gradient^37–40^, and that sickness absence in musculoskeletal disorders is associated with occupations with high physical workload which is consistent with the present study results^37^.

The modifying effect of CRF on sickness absence due to musculoskeletal disorders varied between different occupations. Having high CRF (> 42 ml·min^−1^ kg^−1^) had a beneficial association to sickness absence risk in the main model (not moderated by occupation) and for *shop staff, assemblers, machine operators and related, assistant nurses and home care, construction craftsmen and related trades,* and *construction workers,* most of which are occupation related to higher workload*.* This is similar to a study including male Norwegian conscripts, where conscripts in the low CRF group had a 29% higher risk of musculoskeletal sickness absence 5 to 15 years later^24^. However, for *office assistants, mobile plant operators and vehicle drivers, heavy truck and lorry drivers,* and *cleaners*, a high CRF were either not associated or counterintuitively associated with an increased risk for sickness absence due to musculoskeletal disorders. This highlights the complexity behind sickness absence and indicates that CRF, may in some situations impact sickness absence due to musculoskeletal disorders. For instance, a construction worker with back pain will likely not be able to perform heavy lifting regardless of CRF level.

**Sickness absence due to cardiorespiratory diagnoses** and CRF are links to the functionality of the heart, lung, and their circulating systems. Occupations with greater physical workload exhibited a heightened risk for sickness absence, in the present paper, in line with Swedish research demonstrating a 46% higher prevalence of respiratory disease among a*gricultural workers* compared to m*etal workers*. Moreover, *industrial workers, food industry workers*, and *painters* experienced an increased risk for respiratory disease, which age and smoking habits did not account for, but may be connected to other occupational air pollutants^41,42^. Other studies have reported the importance of CRF in occupational groups with high physical workload, whereas those with low fitness have more adverse events in cardiovascular disease and mortality^19,43^. Our study unveiled a robust moderating impact of CRF across nearly all occupational groups. This finding is relevant given that previous studies have revealed that occupations with low educational attainment generally entail more physical exertion, increasing the likelihood of exceeding the recommended mean aerobic workload during an eight-hour work day (33% of maximal capacity)^9,44^. Which relates to that blue-collar workers with higher CRF have earlier exhibited a lower relative aerobic workload and decreased risk of mortality from ischemic heart disease^17,19^.

This study is partly consistent with the concept known as the 'Physical activity paradox', especially for musculoskeletal and cardiorespiratory sickness absence. The paradox underscores the negative health impact of occupational physical activity compared to leisure-time physical activity. However there could also be other things impacting, such as a range of work-related factors, encompassing the psychosocial and physical work environment, stress, and shift-work^45–47^. Additionally, variations in lifestyle-associated factors that has not been considered in the present study, such as alcohol consumption, could also contribute to this phenomenon^9,48,49^.

We did not have data on many exposure variables of relevance for the studied outcomes, including work conditions or exposure to dirt and dust, dampness, noise, solvents, and problems with lighting or temperature, which could have explained variation in sickness absence between occupational groups. Another limitation was that we did not measure change in occupational group and its effect on sickness absence but used occupation at the time of the HPA, which possibly could dilute the associations between occupational groups and sickness absence. Another limitation of the study is the long time interval between CRF and diagnose-based sickness absence. This could mean that some of the associations observed in the study are due to other factors that have changed over time, rather than to CRF itself. The study may be subject to selection and information bias due to the inclusion of data from multiple registries with varying levels of quality and standardization. A strength was that both mean physical workload from statistics Sweden and METs from Tudor-Locke et. al., supported the definition of variation in self-reported physical workload in the present paper^25^. Another strength was the large study cohort including several occupational groups with a variation in physical workload. Further, the sample was relatively homogenous concerning education, making comparisons between occupational groups less affected by socioeconomical differences, however this also means that we cannot generalize to occupational groups with higher education levels. Two final strengths include an objective assessment of CRF and the use of register-based sickness absence.

Concluding, occupations with a higher physical workload were associated with higher work-related sickness absence. However, higher CRF seemed to moderate some of the risk, especially for sickness absence due to musculoskeletal and cardiorespiratory causes, but not for psychiatric causes. The study results encourage community and workplace interventions to both consider level of physical workload and to maintain and/or improve cardiorespiratory fitness for a lower risk of sickness absence, especially in occupations with high physical workload.

## Methods

Data was retrieved from the HPI Health Profile Institute (HPI) database containing data from Health Profile Assessments (HPAs) carried out since the end of the 1980s in companies connected to occupational or other health services in Sweden. Participation was voluntary and free of charge for the employee. An HPA is a person-centered dialogue that includes self-reported lifestyle habits and perceived health and symptoms. It also includes metabolic and physiological measurements, such as CRF testing. All individual data from the HPA is subsequently recorded in the central database. Health Profile Institute is responsible for the database and software development, standardization and development of methods used, and education of the HPA coaches that perform the HPAs. Additional individual level data was obtained from National registers (Statistics Sweden and Swedish Social Insurance Agency (MiDAS)) and linked by personal identity number. Ethics were granted by the ethics board at the Stockholm Ethics Review Board (Dnr 2015/1864-31/2, 2016/9-32 and Dnr 2019-05711), and the study complied with the Declaration of Helsinki. Informed consent was obtained from the participants prior to participation in the HPA.

**Figure 3:**
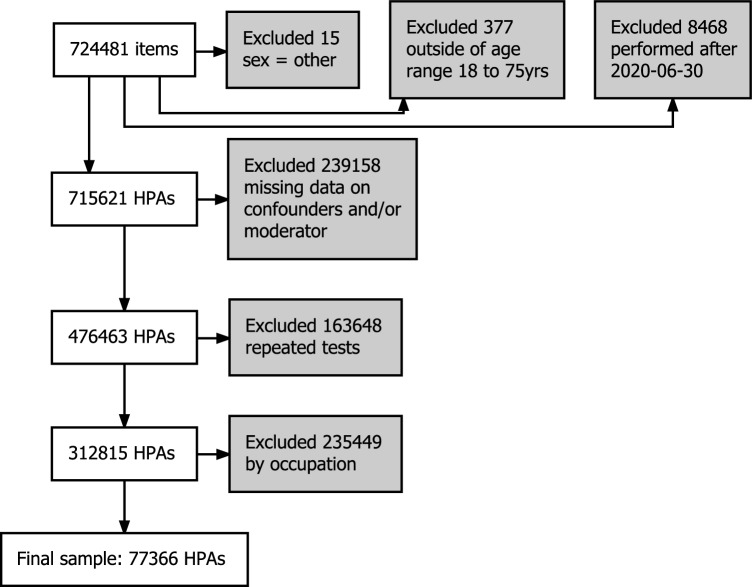
Flow diagram for data cleaning procedure.

Data spans between 1988 and 2020, with the later interval restricted by register data (Fig. 1). After the data cleaning procedure shown in Fig. 3, the resulting sample included 77,366 participants (mean age 41.8 years, range 18–75 years, 52.5% women).

### Occupational group (predictor)

Occupation at the time of the HPA was attained from Statistics Sweden and coded according to the Swedish Standard Classification of Occupations 1996 and 2012 (SSYK)^50^, which are based on the International Standard Classification of Occupation^51^. A 4-digit code describes each occupation, where the first digit defines the major group of occupation, the second digit defines the sub-major group, the third digit defines the minor group, and the fourth digit defines the unit group. In the present study, occupational groups based on 3- or 4-digit level were included. The occupational groups were chosen to cover large occupational groups with mainly low educational requirements (occupations from SSYK major level four to nine that does not include occupations requiring university-level qualifications or equivalent), to minimize the influence of variation in socioeconomics between occupations in the analyses(SSYK-codes in Supplementary Table 1). Both female and male dominated occupational groups were included, often referred to as blue- or pink-collar occupations elsewhere. Occupations included were *Office assistants; Mobile plant operators and vehicle drivers; Shop staff*; *Heavy truck and lorry drivers*; *Assemblers*, *machine operators and related; Assistant nurses and home care*; *Cleaners*; *Construction craftsmen and related trades*; and *Construction workers* (SSYK-codes in Supplementary Table). To validate the inclusion of occupational groups with mainly low educational requirements, individual data on length of education was derived from Statistics Sweden and ranged from < 9 years to post graduate. In Table 1, the proportion with short education (≤ upper secondary school) is presented. At the same time a key criterion for selection of occupational groups was a variation in physical workload. This was assessed using self-reported data from the HPA questionnaire, where the participant answered to the statement *My physical work situation is…* with the alternatives *Sitting with some movement, Physically active, Occasionally physically demanding,* or *Occasionally very physically demanding*. The proportion of participants in each of the included occupational groups stating to have *Occasionally physically demanding,* or *Occasionally very physically demanding* were used to rank the occupations from low frequency of physically demanding workload to high frequency of physically demanding workload (data in Table 1). This order was further used in all tables and figures. This order were subsequently externally validated against two external data sources (data in Table 1). First, aggregated data from Statistics Sweden presenting physical workload in workers from the same occupations as the ones chosen in the present study was used. The aggregated data was presented as percentage of employees in each occupation which at least a quarter of the time defined their work to be physically demanding, defined as having to exert themselves so that breathing becomes faster ¼ of the time^52^. Secondly, we used the MET-values from the 2002 Census Occupational Classification System^25^ assigned to each occupation. Correlation of rankings between the occupation based on HPA self-reported data and the aggregated data from Statistic Sweden was 0.64, and between rankings of occupations based on HPA self-reported data and MET-values 0.71 (both spearman correlation rank coefficient), implying a moderate to strong rank correlation.

### Sickness absence (outcome)

Data on psychiatric, musculoskeletal, and cardiorespiratory sickness absence diagnoses were derived from the MiDAS registry and included sickness absence registered as absence of > 14 days. Sickness absence can be granted part-time (25%, 50%, or 75%) of ordinary working hours and up to a certain limit (approximately 80%) of lost income is covered by sickness absence benefits. Part-time sickness absence days were combined with all sickness absence after the HPA within the study period to net days. For example, two days with 50% sickness absence will be counted as one day. International Classification of Diseases version 10 (ICD-10) was used to classify sickness absence due to different diagnoses; sickness absence due to Psychiatric causes (F01-F99), Musculoskeletal causes (M01-M99), or Cardiorespiratory causes (J01-J99, I01-I99). Sickness absence (> = 14 days in F01-F99, M01-M99, J01-J99 or I01-I99) before the HPA was coded as 1 and was used as a control-variable. Sensitivity analyses of which diagnoses to be chosen were made for psychiatric sickness absence. However, there were only small differences in estimates for model 1 and 2 as well as in the CRF moderated analyses between including all F-diagnoses (as in the main analyses) compared to only including diagnoses often called common mental disorders (major depressive disorders (F32–33), phobic anxiety disorders (F40), other anxiety disorders (F41), obsessive–compulsive disorders (F42), and reaction to severe stress and adjustment disorders (F43)).

### Cardiorespiratory fitness (moderator)

CRF was assessed using the submaximal Åstrand cycle ergometer test and relative VO_2_max was calculated as ml·min^−1^ kg^−1^^53^. The test has been validated against directly measured VO_2_max during treadmill running with non-significant mean differences on group level (− 0.07 L·min^−1^, 95% CI − 0.21 to 0.06) and with an absolute error and coefficient of variance similar to other submaximal tests (SEE = 0.48 L min^−1^, CV = 18.1%)^54^. CRF was categorized arbitrarily into < 32, 32–42, > 42 ml·min^−1 ^kg^−1^.

### Covariates

Covariates were included based on a directed acyclic graph (Supplementary Fig. 1). Age and sex were attained at the HPA. Body mass and height were obtained with standard measures and BMI was calculated as kg m^−2^. Calendar time was coded as year 2020 minus the year of the HPA and included as a continuous variable in the analyses. Length of education was assessed from Statistics Sweden and used as a continuous variable in the models. Smoking habits, stress at work, and exercise habits were self-reported by the statements *I smoke* … with the alternatives *At least 20 cig/day, 11–19 cig/day, 1–10 cig/day, Occasionally,* or *Never*; *I experience stress at work…* with the alternatives *Very often, Often, Occasionally,* Rarely, or Never; and *I exercise for the purpose of maintaining/improving my physical fitness, health and well-being* … with the alternatives *Never, Sometimes, 1–2 times/week, 3–5 times/week*, or *At least 6 times/week.* Smoking was used as a binary variable (Daily smoker ≥ 1 cig/day or not), stress at work and exercise were used as categorical variables.

### Statistical analyses

Sickness absence data is often zero-inflated (with excess of zero counts and not fitting any standard distribution) and over-dispersed (large spread in the data). There are several models that can be appropriate for this type of data, for example a zero-inflated Poisson, negative binomial regression, zero-inflated binomial, and a hurdle model^55,56^. To select the most appropriate model for the data, we compared the four abovementioned models using the Akaike information criterion (AIC). A lower AIC indicates a better fit to the data. The hurdle model had the lowest AIC and was therefore selected for the main analyses. The hurdle model indicates when a threshold or a “hurdle” is cleared, with positive counts splitting the data into two parts; (1) the binary logit model where the risk of not having sickness absence (zero counts) after the HPA is modelled—from now called the zero model, and (2) a zero-truncated negative binomial model where the amount of sickness absence days after the HPA is modelled (only including positive counts)—here on called the count model. An offset with time-in-study was used to account for different follow-up times of the HPA. The estimates for the zero model were expressed as odds ratio with 95% confidence intervals (OR 95%CI) and for the count model they were expressed as incidence rate ratios with 95% confidence intervals (IRR 95%CI). All parameters were estimated by maximum likelihood estimation.@@@

We created two hurdle models that were applied for total sickness absence and for the four different sickness absence categories. Model 1 (M1) was adjusted for age, sickness absence incidence before the HPA, and calendar time. Model 2 (M2) was additionally adjusted for sex, BMI, smoking, stress at work, exercise, and length of education. In Tables 2 and 3, M1 and M2 is stratified by occupational group and sickness absence diagnosis, while in Fig. 1 they are stratified only by diagnosis. Further, for Fig. 2, CRF was added to the M2 model to investigate the moderating effect between CRF groups and occupational group. Predictions in Fig. 2 consider the risk of having a sickness absence diagnosis as well as the number of sickness absence days. This was achieved through multiplication of the binary logit predictions (probability of sick leave) with the zero-truncated negative binomial predictions (predicted days of sick leave in those with sick leave). To get the prediction for sickness absence per 10 years, we divided the predicted sickness absence days with the mean years in study and multiplied it with 10. For the prediction, three different model configurations were used. For all these we used an interaction term between occupational group and CRF. A main model between sickness absence and CRF-group adjusted for the same variables as M2-model can be seen in Fig. 2. All statistics and graphics were made with R^57^ and the packages tidyverse^58^, pscl^59^, and ggeffects^60^.

### Ethical approval

Ethics were granted by the ethics board at the Stockholm Ethics Review Board (Dnr 2015/1864-31/2, 2016/9-32 and Dnr 2019-05711), and the study complied with the Declaration of Helsinki.

### Supplementary Information

Supplementary Information.

## Data Availability

The data that support the findings of this study are available from the HPI Health Profile Institute (HPI) but restrictions apply to the availability of these data, which were used under license for the current study, and so are not publicly available. Interested parties can request access to the data from the authors upon reasonable request and with the permission of HPI. For inquiries regarding data access, please contact HPI at peter.wallin@hpi.se."
