# Supplementary material for: Moderating effect of cardiorespiratory fitness on sickness absence in occupational groups with different physical workloads
Source: Sci Rep. 2023 Dec 21;13:22904. doi: 10.1038/s41598-023-50154-9 (PMC10739801; doi:10.1038/s41598-023-50154-9)

Appendix

Table S1. Occupation, SSYK-version, code and count within each SSYK-code.

Occupation	Code version	SSYK-code	n
Office assistants	Ssyk2012	411	3,344
Office assistants	Ssyk2012	422	1,006
Office assistants	Ssyk96	411	2,031
Office assistants	Ssyk96	412	4,555
Office assistants	Ssyk96	419	6,544
Office assistants	Ssyk96	422	2,534
Assemblers, machine operators and related	Ssyk2012	821	1,349
Assemblers, machine operators and related	Ssyk96	724	3,393
Assemblers, machine operators and related	Ssyk96	821	4,561
Assemblers, machine operators and related	Ssyk96	828	4,605
Assemblers, machine operators and related	Ssyk2012	7223	1,159
Shop staff	Ssyk2012	522	1,148
Shop staff	Ssyk96	522	5,259
Assistant nurses and home care	Ssyk96	5132	12
Assistant nurses and home care	Ssyk96	5133	7
Assistant nurses and home care	Ssyk2012	5321	1,234
Assistant nurses and home care	Ssyk2012	5330	492
Assistant nurses and home care	Ssyk96	5132	7,496
Assistant nurses and home care	Ssyk96	5133	4,466
Mobile plant operators and vehicle drivers	Ssyk2012	834	714
Mobile plant operators and vehicle drivers	Ssyk96	832	979
Mobile plant operators and vehicle drivers	Ssyk96	833	1,777
Cleaners	Ssyk96	9122	18
Cleaners	Ssyk2012	9111	719
Cleaners	Ssyk96	9122	3,456

Occupation	Code version	SSYK-code	n
Construction craftsmen and related trades	Ssyk2012	713	616
Construction craftsmen and related trades	Ssyk96	713	6,769
Heavy truck and lorry drivers	Ssyk96	8323	1,004
Heavy truck and lorry drivers	Ssyk2012	8332	770
Heavy truck and lorry drivers	Ssyk96	8323	18
Construction workers	Ssyk2012	712	958
Construction workers	Ssyk96	712	4,373

Figure S1. Direct acyclic graph showing assumed relationships between the exposure (Occupation) and outcome (Sickness absence). Light grey edges are non-measured variables. Green nodes show possible bias.

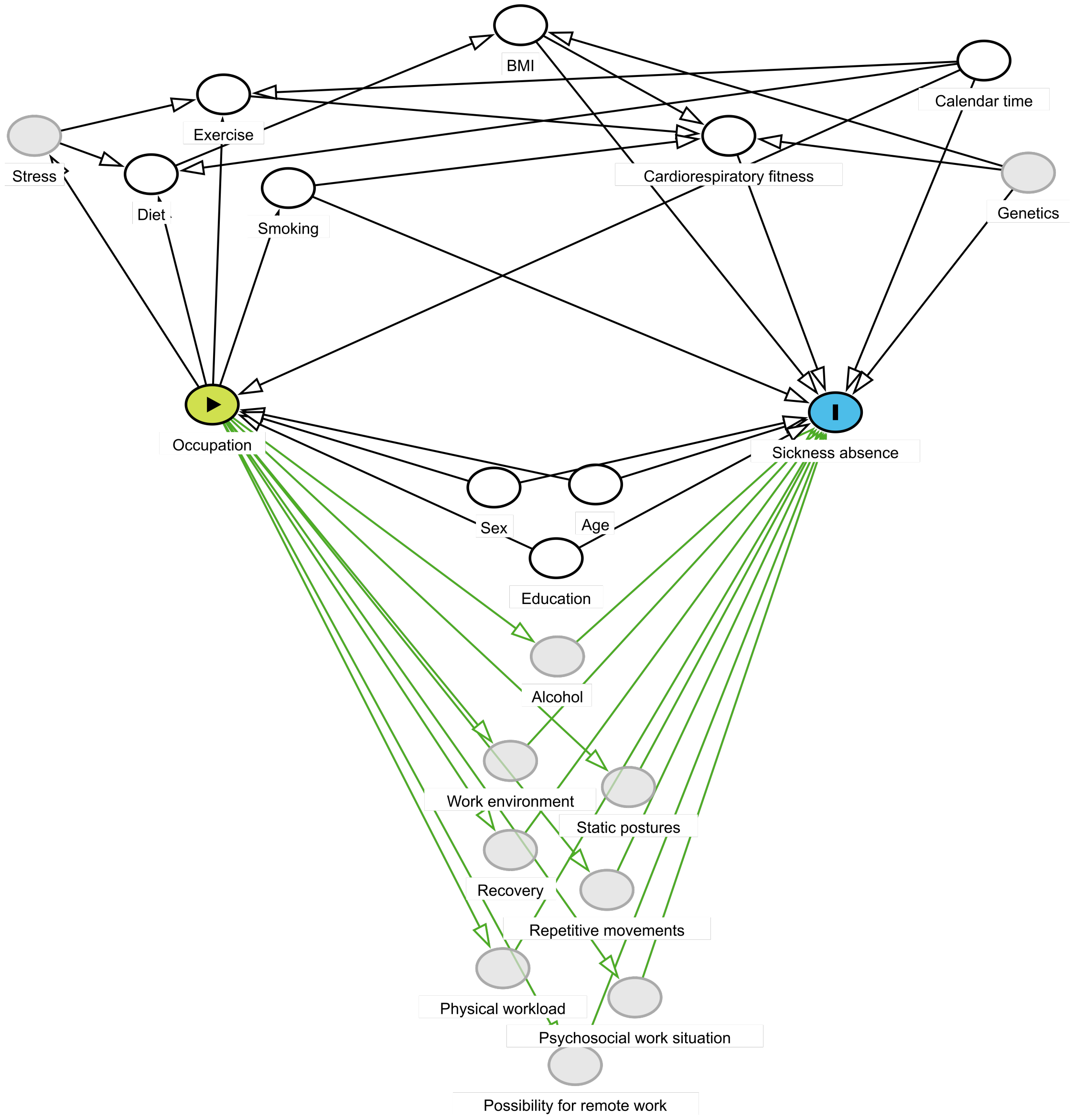

Supplement: Supplementary file 1 — Supplementary Information. [file 41598_2023_50154_MOESM1_ESM.pdf]
